# Eight-year analysis of bullfighting injuries in Spain, Portugal and southern France

**DOI:** 10.1038/s41598-021-94524-7

**Published:** 2021-08-06

**Authors:** Antonio Reguera-Teba, Isidro Martínez- Casas, Pablo Torné-Poyatos, Pedro Hernández- Cortés

**Affiliations:** 1 General Surgery Department, Complejo Hospitalario de Jaén, University Hospital of Jaén, C/Ejército Español, 10, 23007 Jaén, Spain; 2 Juan Ramón Jiménez Hospital, Huelva, Spain; 3 General Surgery Department, San Cecilio University Hospital of Granada, Granada, Spain; 4 Surgery Department, School of Medicine, Granada University, Granada, Spain; 5 Orthopedic Surgery Department, San Cecilio University Hospital of Granada, Granada, Spain

**Keywords:** Anatomy, Health care, Risk factors, Trauma, Epidemiology

## Abstract

Improving knowledge on the epidemiology and analysing the prognostic factors of severity for injuries caused by fighting bulls in Spain, Portugal and southern France. Observational retrospective study including 1239 patients with a reported history of bull horn injuries between January 2012 and November 2019 in Spain, Portugal or southern France. A multiple logistic regression test was used to analyse the prognostic factors of severity and mortality rate of these lesions. The mean accident rate was 9.13% and the mortality rate was 0.48%. The most frequent mechanism of trauma was goring, and the commonest locations of the lesions were thigh and groin. Vascular lesion was found in 20% of thigh/groin gorings. Prognostic factors of severity were vascular lesion, head trauma, fracture, goring injuries and age of the animal. The most reliable prognostic factors of mortality were vascular lesion and goring in the back. Lesions caused by fighting bulls are common in the bullfighting events held in Spain, Portugal and southern France. Although the mortality rate is low, there is a higher morbidity rate, which is conditioned by vascular lesion. All medical teams should include a surgeon experienced in vascular surgery and an anaesthesiologist.

## Introduction

Publications on bull horn injuries are rare in international literature, yet they represent a major health issue in countries with a bullfighting tradition. Bull horn injuries are unique and distinguishable from any other type of lesion. Their incidence is higher in those geographical areas and societies where cattle coexist with people on a daily basis and in those countries with a bullfighting tradition, such as Spain, Portugal, Southern France and Latin American countries^1^. Bull horn injuries caused during the fight of fighting bulls have high morbidity and mortality rates and usually require specialised care.

**Figure 1:**
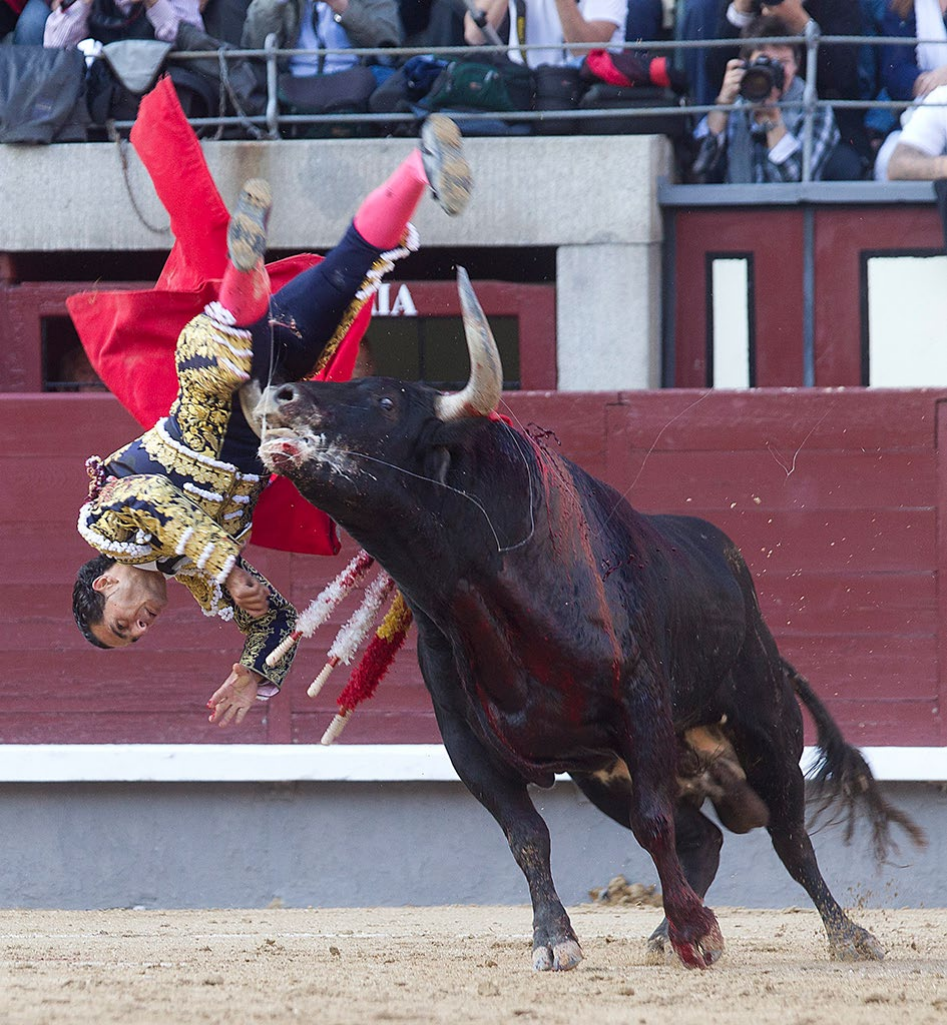
When charging, the bull lowers its head by means of a neck flexion and then extends its neck, introducing one or both horns into the subject’s body (usually in the thigh or groin). This movement creates the first trajectory of the wound. After that, the animal tries to disengage its horn(s) making a rotational movement known as “derrote”, which creates additional wound trajectories. The subjects usually fall on their faces, which implies a risk of head and neck trauma. Courtesy of Carlos Ilián. El Mundo.

**Figure 2:**
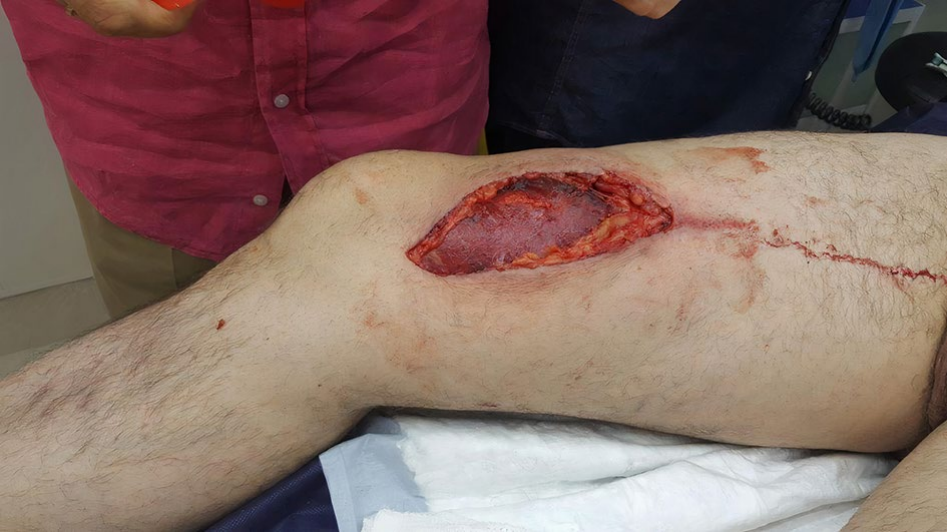
Bull horn injury on thigh. Courtesy of Dr. Enrique Crespo. Clínica Ruber. Madrid.

Deep knowledge of the mechanism of bull horn injury is vital to fully understand the complexity of these wounds. When charging, the bull lowers its head by means of a neck flexion and, once it engages its victim with one or both horns, it extends its neck applying a great force at the point of entry of the horns, which is also a consequence of the mass and acceleration of the animal^2^. As the bull lifts its subject from the floor, it makes a circular movement with its head, making the subject spin around the horns with all their weight on them, causing extensive tissue damage (Fig. 1). Due to the height and manner in which the animal charges, it is most frequent for these lesions to be produced in thighs, groin, perineum, and abdomen. They may become more severe through vascular and/or visceral injuries^3^ (Fig. 2).

Bull goring injuries can be classified into minor traumas, blunt wounds, and penetrating wounds. Penetrating wounds normally have several internal trajectories and a high level of contamination. It is also possible for them to become more severe due to polymicrobial infections and even tetanus. Sheathed goring injuries are considered blunt trauma. In this type of injury, the integrity of the skin is preserved due to its elasticity, but there is a risk of severe deep tissue damage.

The individual prognosis of patients does not only depend on the characteristics of the injury^4^ (Table 1), but also on the scene of the accident (where we can observe a great variability in the structure of the bullring), weather conditions, location of the infirmary, training of the medical team, and distance to the nearest hospital. The share of patients who can be treated in bullring infirmaries and of those who must be transferred to hospitals to receive urgent treatment of the lesion or follow-up remains unknown to us.

**Table 1:** Miñano et al.^9^ bullhorn injuries classification.

Type of injury	Main lesion	Prognosis	Therapeutic approach
A	Wound Focused tip impact Contusion	Minor	Minor surgery
B	Non-penetrating injuries to the musculoskeletal system	Less serious/serious	Immobilisation Analgesics/pain killers Transfer to hospital
C	Goring without involvement of vital organs	Less serious/serious	Major surgery Transfer to hospital
D	Goring with involvement of vital organs Trauma with severe neurological impairment	Serious/very serious	Stabilisation Emergency surgery Control of bleeding Urgent transfer to hospital

According to the Spanish Ministry of Culture and Sports, around 1600 bullfighting events are held yearly in Spain^5^.

We can find different kinds of bullfighting events, with the main ones being the following:

- Bullfights. Bullfighting events where normally six toros bravos (fighting bulls) of 4–6 years of age are to be fought.
- Rejoneos. Bullfighting events where bulls or bullocks are fought on horseback.
- Novilladas. Bullfighting events where the bulls fought are younger and lighter than those used in bullfights and rejoneos.
- Becerradas. Bullfighting events where professionals or amateurs fight bulls younger than 2 years of age.
- Encierros. Traditional festivities consisting in amateurs running in front of the bulls to drive them into the bullring. A famous example is the San Fermin’s festivity in Pamplona.
- Forcados, which is a way of bullfighting, typical from Portugal, where a group of men hold and immobilise a bull.

The main actors participating in these events are listed in Table 2.

**Table 2:** Actors participating in bullfighting festivals.

Actor´s designation	Type of bullring	Activity	Type of event
“*Matador*” (bullfighter)	1st, 2nd and 3rd	Fight and kill the bulls	Bullfight *Novillada* *Becerrada*
*“*Banderillero” (bullfighter assistant)	1st, 2nd and 3rd	Assistance in bullfighting and harpoon	Bullfight *Novillada* *Becerrada*
“*Picador*”	1st and 2nd	Harpoon the bull while riding a horse	Bullfight Some types of *Novilladas*
“Rejoneador”	1st, 2nd	Fight and kill the bulls while riding a horse	*Rejoneo*
“*Forcado*”	1 st,2st and 3rd (the final event in a typical Portuguese bullfight)	A group of men that performs the pega de cara ("face catch")	*Bullfight*
Public of the show	1st, 2nd and 3rd	Run ahead the bulls	*Encierro, Becerrada*
Others (stockbreeders, slaughterers, …)	1st, 2nd and 3rd	Assistance in bullfighting	Bullfight *Novillada* *Becerrada* *Encierro*

The Spanish bullfight regulation provides a legal framework to regulate the preparation, organisation and development of bullfighting events. It was established in Spain in 1992 by the Royal Decree 176/1992. This regulation determines, among other things, the required minimum weight of the cattle for every kind of bullfighting event, the requirements for the infirmary of the bullring, and the composition of its medical team according to the type of event and class of the bullring (Table 3).

**Table 3:** Composition of medical team for the infarmary of the bullrings.

Tipo de festejo	Categoría	Peso del animal	Personal sanitario	
*Corrida de toros* *Rejones*	1	460	2 cirujanos 1 anestesista 1 enfermero	
	2	435		
	3	410		
*Novidalladas sin picadores*	1	< 540		
	2	< 515		
	3	< 270		
*Otros*	Sin especificar		1 cirujano 1 médico 1 enfermero	

Composition of medical team for the infarmary of the bullrings.

Bullrings are classified in 1st, 2nd and 3rd class. Those of first and second class are equipped with surgical infirmaries and generally located in provincial capitals and cities. However, third class bullrings are mostly smaller buildings, usually temporary or mobile, located in villages and lacking an infirmary and/or an operating room. In fact, their assistance service is usually limited to an ambulance. Ironically, Vaquero et al. (2008)^6^ published that lesions are more frequent in villages and 3rd class bullrings, mainly affecting non-professional participants in events like *encierros* and *becerradas*.

Knowledge on bull horn injuries is insufficient as individual experience requires several years before conclusions can be drawn. Additionally, publications on this topic are rare in international literature and existing ones are focused on clinical cases or case series limited to a given hospital^7–10^. As far as we know, only one study on prognostic factors in fighting bull horn injury has been published^11^. It included a series of 204 injured patients and was published 30 years ago.

Thus, we present a study including 1239 injured patients with the aim of improving the knowledge on epidemiology and analysing the prognostic factors of severity for injuries caused by fighting bulls in Spain, Portugal and southern France. The goal of this study is to improve decision-making during on-site care and to suggest modifications of the bullfighting regulation in force, specifically of the minimum requirements for infirmaries and medical team composition in bullrings.

## Methods

We carried out an observational retrospective study including 1239 patients with a reported history of bull horn injuries between January 2012 and November 2019 in Spain, Portugal or southern France. The aim of this study was to analyse the incidence, associated factors, and prognosis of bull horn injuries.

### Database search

Accident reports published routinely in the Spanish bullfighting journals *Aplausos* (Spain, ISSN 1135-4089) and *6 Toros 6* (Spain, ISSN 1135-7304) were used as sources of information about the injured patients. These reports were written by the respective chief surgeon of the bullring medical team, who always specializes either in general and digestive surgery or in orthopedic surgery and traumatology. The journals publishing them include weekly chronicles of bullfighting events held in Spain, Portugal and southern France, which prompted the designation of such geographical area for the study. According to the current regulations in place, the chief of the bullring medical team must provide a written report of any medical or surgical service provided in the bullring and in relation to the bullfighting event held. The report must be made regardless of whether the injured patient is any kind of professional matador (Table 3) or part of their team, a breeder, an ancillary staff member, part of the audience, or an aficionado. They also must include the name, age, sex, and occupation of the injured patient, as well as the type of lesion, the anatomical location, the prognosis, the type of care provided and whether there was need for a hospital transfer.

As this was a retrospective and documental study, it was not possible for us to collect the informed consent from all the patients included in the series. Due to this, we anonymized the used database accordingly in order not to be unethical and infringe the right to privacy of the patient. Informed consent was waived by a provincial ethics committee and the Jaén General Hospital ethics committee. Informed consent was obtained to publish the image (Fig. 1) in an online open access publication.

### Inclusion and exclusion criteria

The series comprised all adults injured by fighting bulls in different bullfighting events held in the designated area during the 8-year period of the study and whose medical reports including the relevant information were published. Exclusion criteria were minor patients, incomplete medical reports (lacking type of lesion, anatomical location and/or prognosis), and lesions produced during bullfights but not by the animal. 12 cases were excluded due to poor medical reports.

### Assessment

Variables analysed were age and sex; bullring category (first, second or third class); scene of the accident; type of event (bullfights, novilladas, rejoneos, other); age of the animal (4–6, 3–4, 2–3, < 2 years of age) and weight of the animal (bulls: > 460, 460–435, 435–410 kg; bull-calves: 540–515, 515–270, < 270 kg); category of the injured professional (matador, banderillero, rejoneador, picador, other); type of lesion (goring, contusion); main lesion (fracture, penetrating wound, head trauma, sprain, muscle tear, dislocation, other); anatomical location of injury (thigh-groin, leg, upper extremity, thoracic-axillary, maxillofacial, back, perineum, abdomen, neck, gluteus); occurrence (and type, when applicable) of vascular lesion; other associated lesions; initial prognosis (mild, moderate, severe, serious, guarded); need for a hospital transfer and mortality rate.

The prognosis of injuries included in the medical report of the bullring infirmary corresponds to the estimated absence days until the injury is healed. Lesions with a mild prognosis require less of 15 absence days, moderate prognosis 15 to 30 absence days and those with a severe prognosis, more than 30 days. Injuries with guarded prognosis are serious and have no estimate regarding their development and outcome, which may vary depending on eventual complications. Those injuries with a serious prognosis are extremely severe and life-threatening for the subject.

### Statistical methods

The statistical analysis was carried out through the SPSS software for Windows, version 23.0 (IBM SPSS Inc., Chicago, IL). The aim of this study was to identify prognostic factors related to the severity and mortality rate of bull horn injuries. The Shapiro–Wilk test was used to check the normality of the quantitative variables.

A multiple logistic regression test was used to assess the correlation between all variables and mortality. A stepwise binary logistic regression test was used to predict the prognosis of the injury. As prognosis was a variable of five modalities, it was dichotomized into favorable (mild or moderate) and poor prognosis (severe, grave and/or guarded). The Hosmer–Lemeshow statistical test was used to assess the goodness of fit of the regression model. The confidence interval was 95%. A value of *p* < 0.05 was considered significant.

## Results

**Figure 3:**
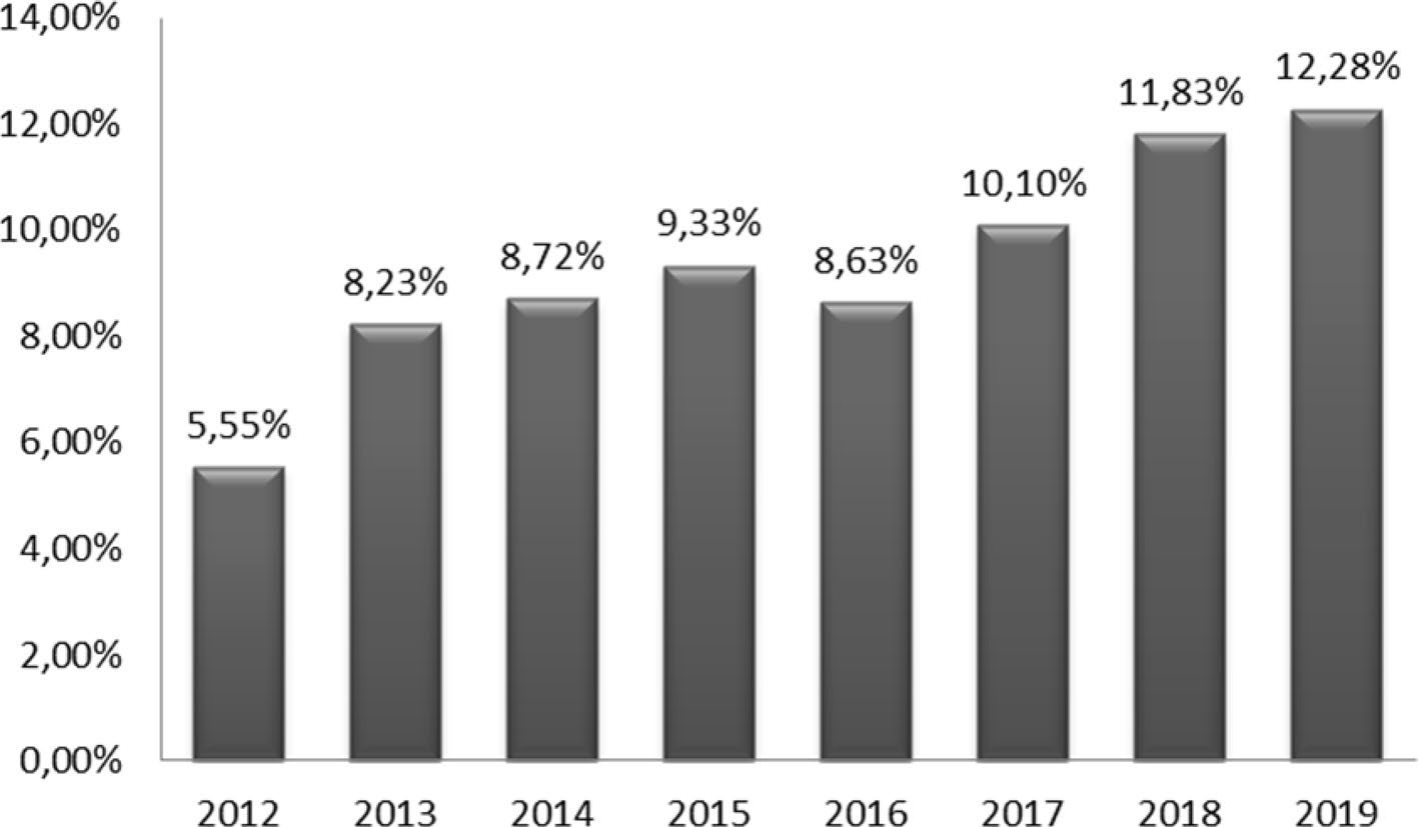
Accident rate (injured patients/ total of events held) × 100.

We found 1239 lesions caused by bull horn in a total of 13,556 events held during the study period. The mean accident rate was 9.13%. The rate of injured patients in each event and year is shown in Fig. 3. The highest and lowest accident rates took place in 2019 (12.28%) and 2012 (5.5%), respectively. A progressive increase in the accident rate during the 8-year period of the study was observed.

The mean age of the series was 35.4 ± 3.24 years (range 18–54), with a male–female proportion of 98.5:1.5. Six hundred thirty-eight accidents happened in 3rd class bullrings (51.5%), 391 in 1st class (31.6%) and 146 (11.8%) in 2nd class ones.

Six hundred seventy-nine lesions (54.8%) happened in bullfight*-*like events; 446 (36%) in novilladas; 68 (5.5%) in rejoneos and 59 (3.1%) in other minor events.

The accident rate by type of event is highest in bullfights (18.75%), followed by novilladas (10.51%) and rejoneos (4.51%).

Matadors were subject to most of the accidents (900 cases; 72.6%). They were followed in frequency by *banderilleros* (199 cases; 16.1%); *rejoneadores* (66 cases; 5.3%); picadors (19 cases, 1.5%); and other professionals (58 cases, 5%).

**Figure 4:**
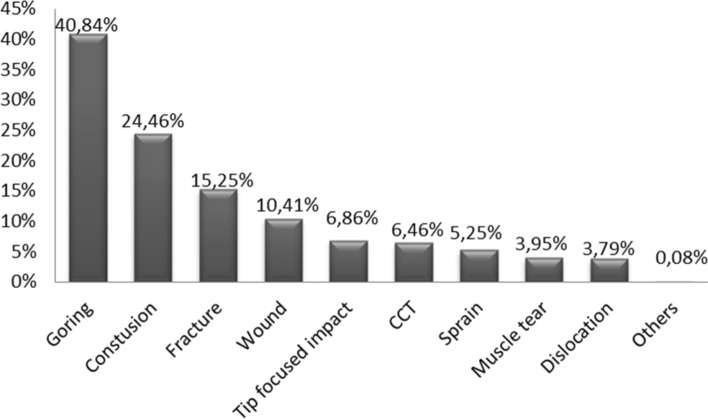
Distribution in percentage of cases according to the type of main lesion.

The commonest type of lesion was goring, which was present in 506 patients (40.84%). Blunt injuries were to be found in 303 patients (24.46%). A total of 298 patients sustained multiple lesions, of whom 65 (5.24%) fit into the criteria of severely polytraumatised patients (having multiple life-threatening injuries). The distribution of the main injuries is shown in Fig. 4.

**Figure 5:**
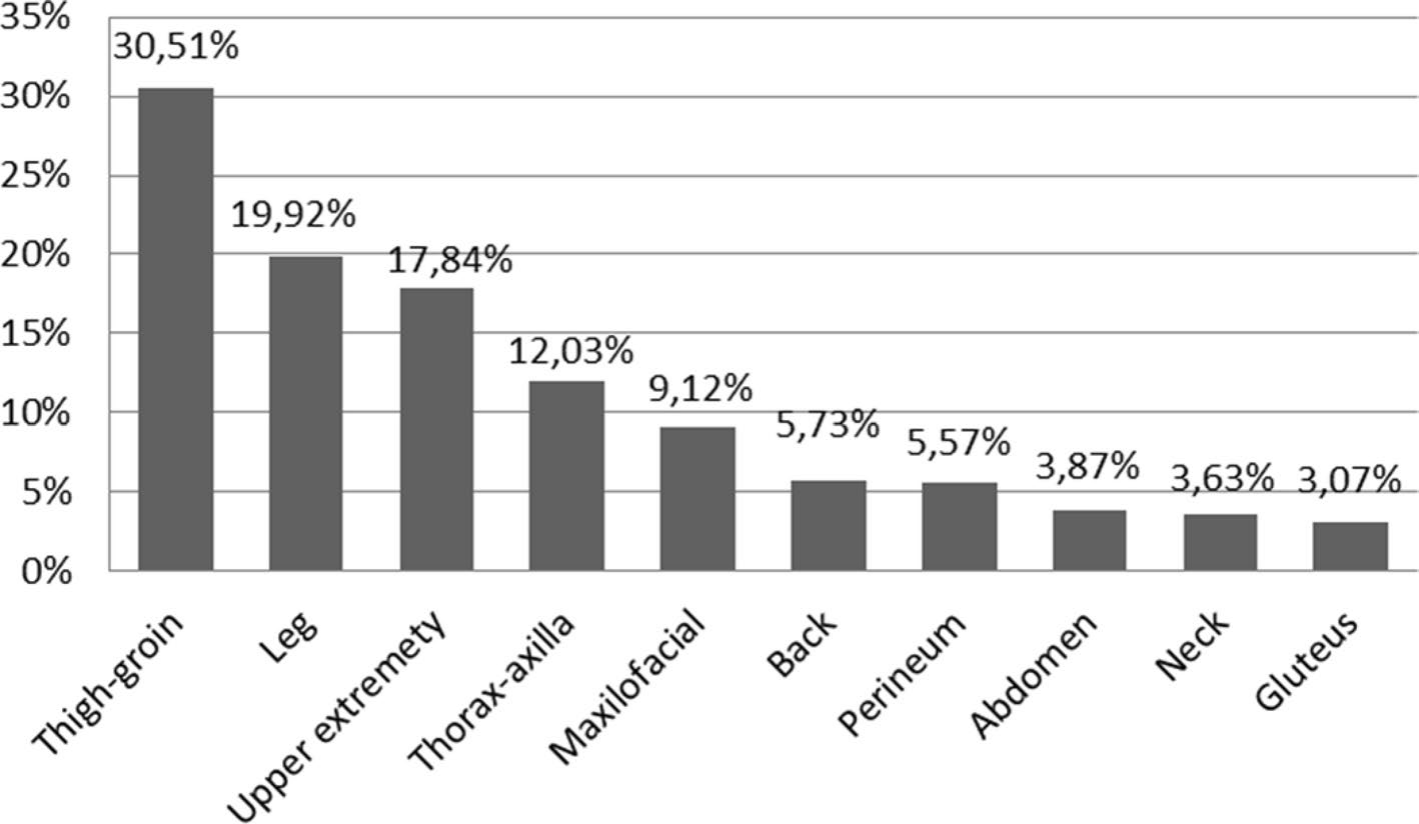
Distribution in percentage of the injuries by anatomical location.

The most frequent anatomical location of injuries was the thigh and groin area (378 patients; 30.51%) followed by the leg (222 patients; 17.92%). The distribution of injuries by anatomical location is represented in Fig. 5.

76 vascular lesions were recorded in the series. Three of these lesions resulted in immediate death (they were located in the chest, damaging great vessels, and in one case, also the heart). The remaining 73 vascular lesions were eligible for surgical repair, 70 of them were located in the groin area, 2 damaged the arteries and iliac veins (and required a laparotomy on-site) and 1 the axillary artery. Thus, vascular involvement was present in 19.04% of injuries located in thigh and groin. All patients with vascular lesion who underwent vascular repair survived. One of them suffered the amputation of a lower extremity. There is a higher probability that vascular lesions will occur in first class bullrings (7.9%) than in second (4.8%) or third class (5%) ones. Nonetheless, no statistically significant difference was appreciated (Chi-squared; *p* = 0.130).

Fifty-one percent of accidents (632 cases) were caused by 4–6-year-old bulls, 40% (495 cases) by 3–4-year-old bulls and the remaining 9% by calves younger than 3 years of age.

The prognosis for this lesion was classified as mild in 314 cases (25.3%), moderate in 242 (19.5%), severe in 327 (26.4%), serious in 43 (3.5%) and guarded in 301 (24.5%). Six patients (0.48%) died as a result of the injury.

Ruling out cases of guarded prognosis and severe injuries requiring urgent hospital transfer, 71% of the injured patients treated in our study could benefit from a definitive treatment carried out in a bullring infirmary.

Results of the multiple logistic regression test concerning prognosis are shown in Table 4 and those concerning mortality rate in Table 5.

**Table 4:** Results of multiple logistic regression (prognosis).

Variables	Odds ratio	Max	Min	
Type of lesion	Vascular lesion	43.970	5.938	325.581
	CCT	9.769	5.139	18.572
	Fracture	7.810	4.988	12.230
	Dislocation	5.784	2.844	11.764
	Goring	6.182	4.291	8.908
Age of the animal	< 2	1.356	0.313	5.879
	2–3 y	0.918	0.568	1.482
	3–4 y	0.568	0.433	0.747
	4–6 y	Reference category		
Category of the injured professional	Bullfighter	Reference category		
	*Banderillero*	1.350	0.946	1.925
	Picador	1.641	0.571	4.714
	*Rejoneador*	2.269	1.250	4.117
	*Other*	3.335	1.547	7.188

**Table 5:** Resultados de la regresión logística (mortalidad).

Variable	Odds ratio bruta	IC para OR 95%		p_valor
		Lim. Inf	Lim. Sup	
Temporada				0.797
Categoría de la plaza				0.231
Tipo de festejo				0.627
Categoría del lesionado				0.318
Sexo				0.999
**Cornada**	**4424**	**1192**	**16,423**	**0.026**
Puntazo				0.997
Herida				0.996
Constusión				0.974
Varetazo				0.998
Fractura				0.892
Luxación				0.998
Esguince				0.997
TCE				0.997
Rotura muscular				0.998
Musloingle				0.315
Pierna				0.396
Periné				0.997
Glútea				0.998
**Tóraxaxila**	**3707**	**1102**	**12,464**	**0.034**
**Abdomen**	**13,364**	**3877**	**46,059**	**0.000**
Cara				0.996
MMSS				0.995
Cuello				0.400
Espalda				0.125
Edadaños_cat				0.162
PesodeltoroKg				0.320
PesodelnovilloKg				0.804
**Lesión vascular**	**91,825**	**19,701**	**427,990**	**0.000**
Politraumatismo				0.227

Values in bold indicates significance p<0.05.

The only two risk factors for a statistically significant lethal injury are a vascular lesion or a back injury (multiple logistic regression; *p* < 0.001 and *p* = 0.023, respectively) (Table 6). Patients sustaining a back injury or vascular lesion are, respectively, 11 and 128 times more likely to die than patients whose injuries do not affect any great vessels and are found in other anatomical locations.

**Table 6:** Results of logistic regression multiple (mortality rate).

Variable	Odds ratio	Max	Min	*p*-value
Back injury	10.623	81.261	1.389	0.023
Vascular lesion	128.825	698.820	23.540	0.000

Ninety-one percent of injuries were inflicted by bulls older than 4 years of age. Thus, the animal being younger than 4 years of age has proven to be a protective factor (OR = 1.356, 95%, CI 0.568, 0.747). Particularly, the risk to show a severe, grave and/or guarded prognosis is reduced to 43.2% when the animal is a bull-calf (2–4 years of age) instead of a bull (4–6 years of age). Despite most serious injuries happening in first class bullrings, there was no statistically significant difference between bullring class and prognosis of the lesion (Chi-squared; *p* = 0.288) (Table 7). There was also no statistically significant difference between prognosis and type of event (Chi-squared, *p* = 0.055) (Table 8), even when more injuries were found to be occur in bullfights than in novilladas.

**Table 7:** Contingency tables (Chi-square Ҳ2).

	Prognosis					Total
	Minor	Less minor	Serious	Very serious	Cautius	
**Class of rings**						
1	26.1%	16.6%	27.6%	4.6%	25.1%	100.0%
2	29.7%	20.0%	20.7%	4.8%	24.8%	100.0%
3	25.6%	20.2%	26.9%	2.2%	25.0%	100.0%
Total	26.3%	19.0%	26.4%	3.4%	25.0%	100.0%
1	102	65	108	18	98	391
2	43	29	30	7	36	145
3	161	127	169	14	157	628
Total	306	221	307	39	291	1164

Contingency tables ( Chi- square Ҳ2 ). Prognosis and ring’s category ( p= 0.288)

Prognosis and ring’s category (*p* = 0.288).

**Table 8:**
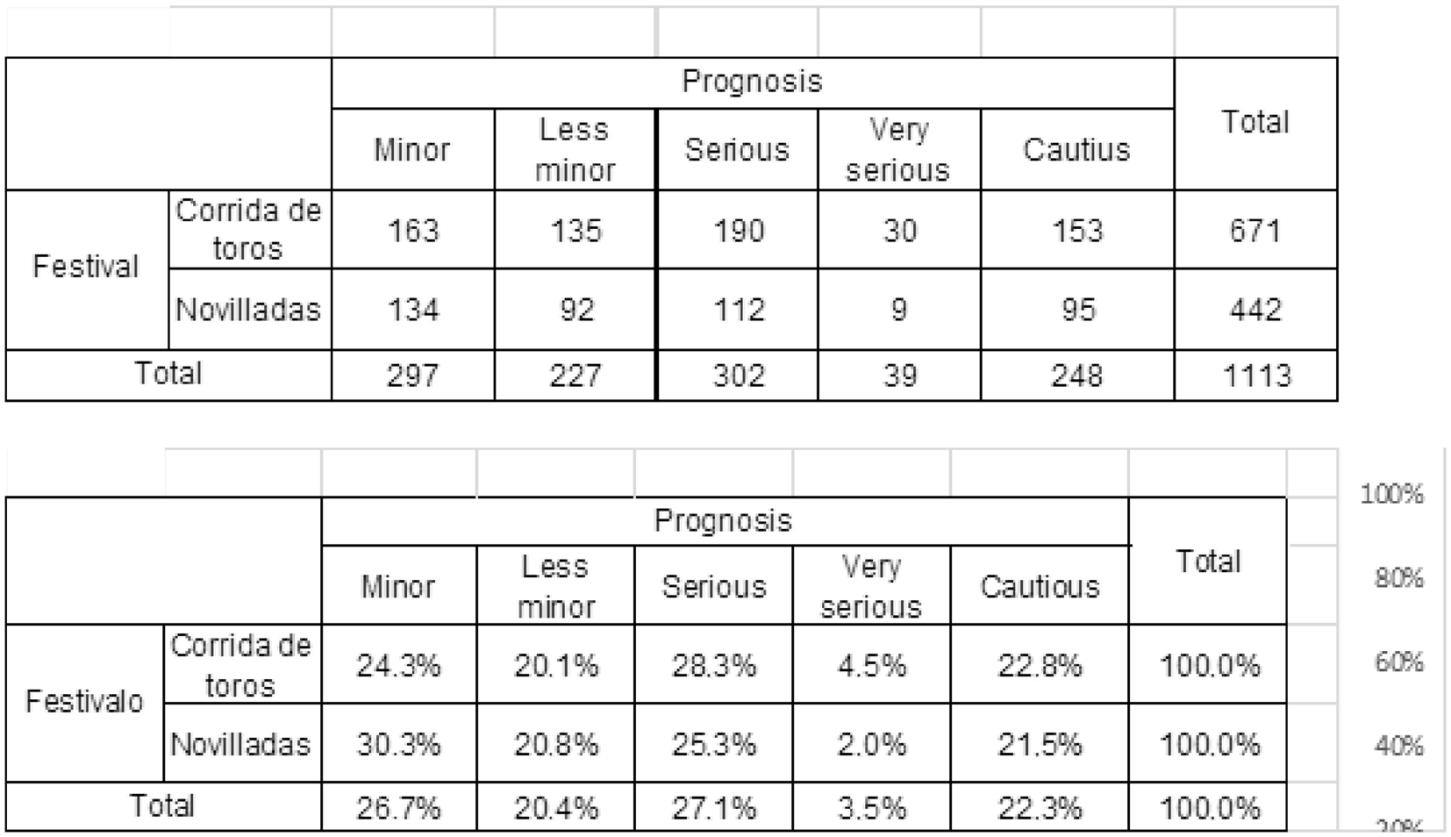
Contingences table.

Chi-square (Ҳ2). Prognosis and type of festival (*p* = 0.055).

The 6 deaths (0.48% mortality) registered in the study were of three matadors, two *forcados*, and a *recortador*. The main lesions among deceased matadors were chest goring with great blood vessel injury in two cases and a severe spinal cord injury in the remaining case. The cause of death in *forcados* was blunt abdominal trauma and hypovolaemic shock. The *recortador* died as a result of a chest goring, with cardiac injury. Four of the deaths registered in the study happened in third class bullrings and one in a second-class bullring. No deaths occurred in first class bullrings.

A total of 31 gorings on chest and underarm were recorded in the study: 20 of them occurred in first class bullrings, 2 in second class and 9 in third class ones. Hence, the mortality rate of chest injuries was 9.67%.

Forty-eight abdominal trauma were recorded. There were 27 open abdominal injuries and 21 blunt abdominal traumas. Thirty patients underwent laparotomy. Fifteen of these operations took place in first class bullrings, five in second, and eight in third class ones. There were no deaths among the patients surgically treated at the bullring infirmary, but two *forcados* that suffered blunt abdominal trauma and were transfer to hospital for delayed laparotomy died.

## Discussion

Publications on bull horn injuries are rare in international literature and existing ones are focused on clinical cases or case series limited to a given hospital.

One of the most important aspects of our paper is that it is the first study on prognostic factors in fighting bull horn injuries to be published in the last 30 years. The most recent article on this topic was published in 1990 by Monferrer-Guardiola^11^. Our study probably gathers the widest series ever published, as it included more than 1200 injured patients.

The main epidemiological contributions of our study can be summarised in the fact that, even though the mortality rate of this kind of accidents is low (0.4%), the accident rate is significant and almost one victim for every 10 events is to be expected (accident rate: 9.13%).

The commonest profile of patients was that of a male of around 30 years of age, generally a matador or bullfighting professional, who suffered a penetrating injury (present in 40% of the cases recorded in the study) located in the thigh and groin area. 5.24% of victims fit into the criteria of polytraumatised (having life-threatening injuries). Prognosis of the injuries is mild or moderate in almost 50% of cases and up to 70% of injuries are treated definitively in the infirmary of the bullring. These accidents are more frequent in third class bullrings, with no bullring-related significant differences in goring prognosis.

The low mortality rate (0.48%) observed in the study has already been noted by different authors, such as Val Carreres^12^, Vazquez-Bayod, et al. (1997)^13^, Lehman (2003)^14^, and Martínez Ramos et al. (2006)^15^.

This historical downward tendency regarding mortality rate in professional bullfighting events is probably due to the higher quality of care in relation to ATLS (Advanced Trauma Life Support) protocols, the increasingly demanding healthcare requirements stipulated by the legislation in place, and the greater professionalisation of bullfighters.

The accident rate observed coincides with the tendency stated by Chambres^16^, who informs of the need of surgical intervention in one out of fifteen bullfighting events (6.7%) in southern France bullrings. Miñano-Pérez^17^ recorded 317 lesions in 3936 events (8%) in Spain. In our series, the accident rate is somewhat higher and shows a slightly growing tendency during the period of the study. We are not able to explain this trend.

The same typical profile of the bullhorn injured patients is already published by Legido-Morán and Miñano-Pérez^18^. The incidence of injuries in female is anecdotal, as is their active participation in these events. However, other authors^19^ publish that, while the most severe injuries are sustained by matadors and happen during the bullfight in the bullring, the commonest injuries are sustained by aficionados and bullfighting professionals other than matadors (such as stockbreeders or veterinaries), in third class bullrings and minor events such as *encierros* and *becerradas*. The differences between the information previously shown and what we observed in our study are difficult to explain. Probably, the databases used and the variations in events according to the traditions of the different geographical areas could partially justify them.

The injury most frequently published in literature is goring, as we also observed in the study. However, it is worth noting that, in our series, 15% of lesions were fractures and 6% were head injuries. We find these data should be taken into account for the composition and training of medical teams.

Sixty-five patients in our series sustained multiple lesions and fit into the criteria of severely polytraumatised (having multiple life-threatening injuries). When assessing these patients, it is important to consider them as multisystem trauma patients^4,20^. Hence, the medical team must be adequately trained in Advanced Trauma Life Support protocols.

While bull horn injuries can be present in any part of the body, we observed that 49% of injuries were to be found in the lower extremities, mainly in the groin area (31%). As a matter of fact, Ríos Pacheco^21^ and Ruddolf^22^ published that bull horn injuries located in lower extremities represent 57% and 66% of bull horn injuries, respectively. García Marín^23^ also conclude in their systematic review that, according to the largest series, the most commonly affected areas are the lower extremities, especially the thigh and perineum.

Despite having collected 27 open abdominal trauma, early surgical treatment in the infirmary of the bullring resulted in no deaths. This observation justifies the need to have an adequate infrastructure to perform a laparotomy, even in third class bullrings.

As happened in the study of Zamora-Lomeli (2004), chest trauma is the first cause of death in our series, mainly due to heart and great vessel injuries. Thoracotomy is recommended in penetrating trauma, and the urgency and approach to be followed will depend on the haemodynamic status and the injury.

Goring in the back has been shown as a prognostic factor of mortality. The underlying reason is that gorings in the back torso entail a high risk of visceral injuries. Thus, liberal policy of laparotomy should be followed in case of any doubt.

Risk factors for a severe, serious, or guarded prognosis are vascular lesion, head injury, fracture, goring and animal age. The surgeon in the medical team of the bullring must bear in mind that one out of five gorings in the thigh and/or groin areas (around 20%) will be accompanied by a vascular lesion. Although there was no mortality in cases of vascular lesions in the extremities, these require immediate surgical intervention.

Analysis of the information shown encourages us to reflect on the training and qualification of the medical team of the bullring and suggest some modifications of the bullfighting regulations currently in place to diminish the morbidity and mortality rate among patients.

The possibility of assisting polytraumatised patients requires the medical team to be properly trained in Advanced Trauma Life Support. On another front, the common localisation of injuries in the thighs and groin area also calls for the medical team to have a deep knowledge on surgical anatomy of the lower extremity.

Gorings damaging great vessels in the chest are fatal. However, urgent laparotomy in abdominal trauma and immediate treatment of the peripheral vascular lesion are associated to a high survival rate, which is why a vascular surgeon or general surgeon with training in vascular lesion treatment should be required to be a part of the medical team. Even though fractures are sustained by 15% of injured patients, these lesions are not normally life-threatening and, thus, we do not consider the presence of an orthopaedic surgeon to be imperative in the medical team.

Infirmaries of bullrings are a medical-surgical care service similar to that provided in military health care. The commonest procedure consists of an adequate initial stabilisation and a rapid transfer to the nearest hospital centre. New studies are needed to compare outcomes in patients treated in situ and those transferred to hospitals.

The fact that bullring class means no statistical difference in prognosis should promote a better provision of health resources and end the differences in medical team composition in 3rd class bullrings, which are normally the ones farthest from the hospital.

The requirement of the presence of an anaesthesiologist in third class bullrings and minor events only when horses are used (*rejoneo* and picadors) is not logical, as the accident rate in *rejoneo*s is 4.51% compared to 18.75% in bullfights on foot. The anaesthesiologist should be required in the medical team in all bullfighting events. Besides, bullfighting events held in third class bullrings should only allow to fight bulls younger than 4 years of age.

Our study has a number of constraints such as its retrospective design, the limited information offered by medical reports and the fact that we could not include certain factors which are likely to be prognostic, such as medical team composition or distance to a general hospital. Lack of information about transfer to hospital rate, delays in diagnosis and time to surgical intervention, which may influence morbidity and mortality, is another limitation of the study. A regression model based on only 6 deaths as the dependent variable is questionable, especially given the lack of information in the database regarding other potential confounders. However, these limitations do not undermine the value of both the epidemiologic knowledge this study provides and the protocol modifications we suggest. Further successive studies examining hospital stay or complications of these lesions are still needed.

## Conclusions

Fighting bull horn injuries are frequent in bullfighting events held in Spain, Portugal and southern France, although their mortality rate is low.

Prognostic factors of severity are vascular lesion, head trauma, fracture, goring, and age of the animal. The most reliable prognostic factors of mortality are vascular lesion and goring in the back. Twenty percent of gorings located in thigh and groin areas are accompanied by a vascular lesion. The prognosis of the lesions does not vary according to the type of event and/or category of the bullring. Thus, we suggest the presence of a surgeon experienced in vascular surgery and an anaesthesiologist in all medical teams.
